# The Resistance of Narrow-Leafed Lupin to *Diaporthe toxica* Is Based on the Rapid Activation of Defense Response Genes

**DOI:** 10.3390/ijms22020574

**Published:** 2021-01-08

**Authors:** Michał Książkiewicz, Sandra Rychel-Bielska, Piotr Plewiński, Maria Nuc, Witold Irzykowski, Małgorzata Jędryczka, Paweł Krajewski

**Affiliations:** 1 Department of Genomics, Institute of Plant Genetics, Polish Academy of Sciences, 60-479 Poznań, Poland; sandra.rychel-bielska@upwr.edu.pl (S.R.-B.); pple@igr.poznan.pl (P.P.); 2 Department of Genetics, Plant Breeding and Seed Production, Wroclaw University of Environmental and Life Sciences, 50-363 Wrocław, Poland; 3 Department of Biometry and Bioinformatics, Institute of Plant Genetics, Polish Academy of Sciences, 60-479 Poznań, Poland; mnuc@igr.poznan.pl (M.N.); pkra@igr.poznan.pl (P.K.); 4 Department of Pathogen Genetics and Plant Resistance, Institute of Plant Genetics, Polish Academy of Sciences, 60-479 Poznań, Poland; wirz@igr.poznan.pl (W.I.); mjed@igr.poznan.pl (M.J.)

**Keywords:** Phomopsis stem blight, lupinosis, defense response, resistance gene, transcriptome, sequencing, expression

## Abstract

Narrow-leafed lupin (*Lupinus angustifolius* L.) is a grain legume crop that is advantageous in animal nutrition due to its high protein content; however, livestock grazing on stubble may develop a lupinosis disease that is related to toxins produced by a pathogenic fungus, *Diaporthe toxica*. Two major unlinked alleles, *Phr1* and *PhtjR*, confer *L. angustifolius* resistance to this fungus. Besides the introduction of these alleles into modern cultivars, the molecular mechanisms underlying resistance remained unsolved. In this study, resistant and susceptible lines were subjected to differential gene expression profiling in response to *D. toxica* inoculation, spanning the progress of the infection from the early to latent phases. High-throughput sequencing of stem transcriptome and PCR quantification of selected genes were performed. Gene Ontology term analysis revealed that an early (24 h) response in the resistant germplasm encompassed activation of genes controlling reactive oxygen species and oxylipin biosynthesis, whereas in the susceptible germplasm, it comprised induction of xyloglucan endotransglucosylases/hydrolases. During the first five days of the infection, the number of genes with significantly altered expressions was about 2.6 times higher in resistant lines than in the susceptible line. Global transcriptome reprogramming involving the activation of defense response genes occurred in lines conferring *Phr1* and *PhtjR* resistance alleles about 4–8 days earlier than in the susceptible germplasm.

## 1. Introduction

The narrow-leafed lupin, *Lupinus angustifolius* L., is the most economically important species in the tribe Genisteae, which constitutes a part of an early evolved Genistoid clade of the Papilionoid legumes [1,2,3]. As a grain legume crop, *L. angustifolius* is cultivated for its high seed protein content, as well as for its ability to increase soil fertility, improve its structure, and activate soil-bound phosphorus [4,5,6]. Narrow-leafed lupin may serve as a valuable human food ingredient with high nutritional and health properties [7,8,9]. Moreover, it could be considered a good source of essential amino acids, minerals, and dietary fiber [10]. Nevertheless, the major usage of lupin is related to animal feeding, predominantly ruminants, followed by pigs, poultry, fish, and dairy cows [6,11,12]. Thus, during crop domestication, the nutritional properties of *L. angustifolius* were greatly improved.

However, animals grazing on lupin stubble (sheep, cattle, horses, and donkeys) or fed using lupin grain (pigs and poultry) may develop symptoms of lupinosis, a serious and life-threatening hepatotoxicosis disease [13,14,15]. The occurrence of lupinosis has been reported in many countries, including Australia, Germany, New Zealand, Poland, the Republic of South Africa, Spain, and the United States of America [16,17]. The causal agents of lupinosis are the mycotoxins phomopsins A and B, which are produced by the pathogenic fungus, *Phomopsis leptostromiformis* (Kühn) Bubák [18,19], which is currently classified as *Diaporthe toxica* Will., Highet, Gams, and Sivasith. The fungus is a plant endophyte and a pathogen that occasionally causes Phomopsis stem blight [20]. The infection process has a relatively long latent phase (up to several weeks) and toxins are produced after this phase if the fungus successfully colonizes plant tissues [21]. Disease development can be assayed by simple surface evaluations of the lesion’s appearance on senescent stems or via microscopic observations of subcuticular coralloid hyphae structures that develop within infected stems [22,23].

The Australian lupin breeding program targeted Phomopsis stem blight resistance as a key constituent of *L. angustifolius* domestication. It resulted in the creation of a highly resistant breeding line, 75A:258 [23]. This genotype was successfully implemented in Australian breeding programs and is still considered as a reference in phytopathological assays because its resistance to *D. toxica* has never been broken [24,25,26,27,28,29,30]. It was later revealed that *D. toxica* resistance in 75A:258 is conferred by a single dominant allele, subsequently named *Phr1* [29]. A second, unlinked and incompletely dominant allele, conferring moderate resistance to *D. toxica*, was found in cv. Merrit and named *Phr2* [29]. A third allele, providing an intermediate level of resistance between those of *Phr1* and *Phr2*, was identified in the cultivar Wonga and named *PhtjR* [25,27,31]. The *Phr1* gene was supplemented with Ph258M1 and Ph258M2 markers, which were developed with the use of a molecular fragment length polymorphism technique [26]. The *PhtjR* gene was provided with PhtjM4, PhtjM5, and PhtjM7 single nucleotide polymorphism markers [27], as well as with a pair of InDel2 and InDel10 insertion–deletion markers that were developed using high-throughput genome sequencing [25]. The European *L. angustifolius* germplasm collection was recently genotyped with markers tagging *Phr1* and *PhtjR* alleles and phenotyped for *D. toxica* resistance using several strains of fungi originating from Poland and Australia [30]. That study confirmed that the resistance conferred by *Phr1* and *PhtjR* alleles was retained in both field and controlled environment conditions. In Europe, the earliest reports of Phomopsis stem blight disease come from Germany (1880) and Denmark (1892) [32,33]. In central Europe the pathogen is considered dormant, appearing occasionally but it has never caused large-scale epidemics [34,35]. However, the European land climate has been experiencing fast warming in the last few decades, resulting in an increase in the mean temperature equal to *≈*2 °C above the average [36]. Rapid warming, combined with changing precipitation patterns, may favor future incidences of Phomopsis stem blight on lupins in Europe. For comparison, such a scenario is expected for Phomopsis stem canker of sunflower in North America [37].

Despite the significant progress made in the genetic improvements of *L. angustifolius* to Phomopsis stem blight resistance, the molecular mechanisms of this resistance have not been identified. It is only known that resistant accessions hamper the development of the fungus during the latent phase of the infection, preventing the whole plant from being colonized [28,29]. The assembly of *L. angustifolius* pseudochromosome sequences opened novel possibilities for research targeting the transcriptionally active fraction of the genome [38,39]. Analysis of this sequence assembly revealed that *L. angustifolius* genome regions neighboring two major *D. toxica* resistance loci (±400 kbp) encode several candidate homologs of R genes [40], namely, receptor-like kinases. Two such genes were identified for *Phr1* (*TanjilG_11959* and *TanjilG_11965*) and three for *PhtjR* (*TanjilG_26804*, *TanjilG_26805*, and *TanjilG_26824*). This observation supported the formulation of the hypothesis stating that the resistance of 75A:258 and Wonga to *D. toxica* is based on early recognition of fungal activity by receptor-like genes, triggering a multilateral defense. Components of such a response should include the production of antimicrobial compounds, the neutralization of toxins released by the fungus, and an oxidative burst and reinforcement of cell walls. Taking into consideration the aforementioned latent phase in the Phomopsis stem blight disease, we expected the presence of a basic defense in susceptible plants to be launched in response to typical symptoms evidencing colonization of plant tissue by a pathogenic fungus that is, however, expressed too late to efficiently limit and extinguish the infection process. In the present paper, we report the research addressing existing gaps in the knowledge of molecular components of *L. angustifolius* resistance to *D. toxica*. Defense responses of a highly resistant (*Phr1*), a moderately resistant (*PhtjR*), and susceptible *L. angustifolius* accessions were profiled using high-throughput RNA sequencing, followed by differential gene expression profiling and Gene Ontology (GO) enrichment analysis. Genes highlighted by a global transcriptomic assay were then subjected to real-time PCR quantification across time points spanning from the early to late phases of the infection. The hypothetical functional contribution of transcriptionally responsive genes is also discussed.

## 2. Results

### 2.1. Isolates of Diaporthe toxica Represented a Diverse Gene Pool That Is Related to the Host Plant

**Figure 1:**
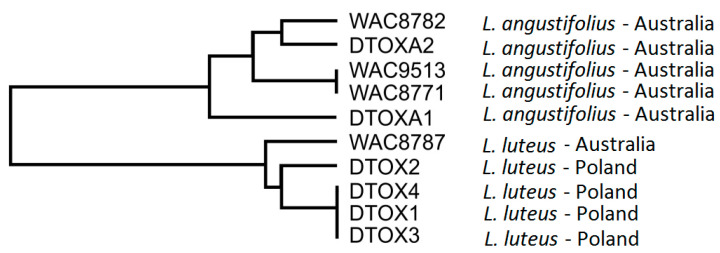
The groups of *Diaporthe toxica* isolates based on Random Amplified Polymorphic DNA data obtained with the use of OPC02, OPG03, OPL05, OPL10, OPJ14, and OPC20 primers; the tree was obtained using the Unweighted Pair Group Method with Arithmetic mean.

Ten *D. toxica* isolates originating from *L. angustifolius* and *Lupinus luteus* plants were analyzed. Random amplification of polymorphic DNA (RAPD) yielded polymorphic products for six primers, namely, OPC02, OPG03, OPL05, OPL10, OPJ14, and OPC20. The internal transcribed spacer (ITS1) fragment sequence differed for three isolates (deletion of one nucleotide in the isolates DTOX1, DTOX3, and DTOX4), whereas the ITS2 fragment was identical for all isolates. Genotype profiling using RAPD-PCR and ITS markers revealed that the genetic diversity of the analyzed *D. toxica* isolates correlates with the host plant and it was not related to its geographic origin (Figure 1). The RAPD scores are provided in the Supplementary Table S1.

### 2.2. PhtjR and Phr1 Alleles Conferred a High Level of Resistance to Diaporthe toxica

The genotyping of lines with markers linked to Phomopsis stem blight resistance alleles confirmed that Wonga and Tanjil were homozygous for *PhtjR* (resistant) and *phr1* (susceptible) alleles; 75A:258 was homozygous for *phtjR* (susceptible) and *Phr1* (resistant) alleles; whereas, Unicrop, Emir, and Baron carried only susceptible alleles for both loci (Table 1).

Controlled environment experiments confirmed the relatively high resistance of lines carrying desired *PhtjR* and *Phr1* alleles; Wonga and 75A:258 were the most resistant; whereas Emir and Baron, lacking both resistant alleles, were the most susceptible.

### 2.3. Resistance to D. toxica Was Associated with Rapid Transcriptome Reprogramming

Plant material sampled from 75A:258, Wonga, and Emir lines was subjected to gene expression profiling using high-throughput sequencing. The protocol of RNA isolation from stems yielded 100 µL of RNA isolate per sample with an average concentration of 310.6 ± 153.8 ng/µL and an average RNA quality indicator value of 7.9 ± 0.5 (Supplementary Table S2). The Illumina NovaSeq 6000 protocol provided, on average, 43.0 mln read pairs per sample (from 30.9 to 62.2 mln read pairs per sample) (Supplementary Table S2). The rate of the read mapping in the reference sequence was from 75.0 to 88.6%. The mean correlation of the count data between biological replications, within experimental variants, was from 0.88 to 0.99. Out of the 35,170 genes analyzed, 2684 had 0 base mean expression, and 3774 had lower than 5. Therefore, the number of genes considered to be expressed was 28,712 (81.64%).

**Figure 2:**
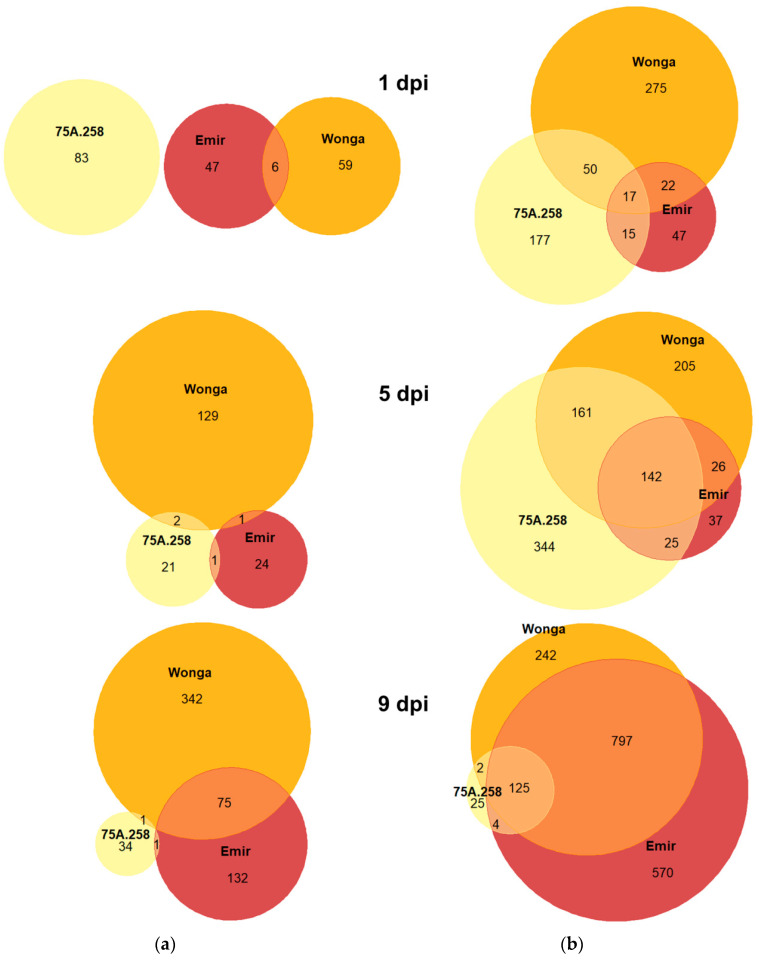
Venn diagrams showing the number of (**a**) downregulated and (**b**) upregulated genes in *Lupinus angustifolius* lines subjected to *Diaporthe toxica* inoculation. The analyzed lines were as follows: 75A:258 (highly resistant, carrying the *Phr1* gene), Wonga (moderately resistant, carrying the *PhtjR* gene), and Emir (susceptible). dpi stands for days post inoculation.

All lines responded to inoculation with *D. toxica* via transcriptome reprogramming (Table 2); however, resistant lines (75A:258 and Wonga) responded more quickly than the susceptible line, Emir. This difference was highlighted by a significantly higher number of genes induced in the first two time points, 1 day post inoculation (1 dpi) and 5 dpi, in resistant germplasm. Moreover, in the first two time points, the overlap of genes upregulated in both resistant lines was considerably greater than the overlap between either the resistant line or the susceptible line (Figure 2).

There was a relatively low percentage of genes that were coherently upregulated in all three lines at 1 dpi (2.8%); however, this value was considerably higher at 5 dpi (15.1%) and 9 dpi (7.1%). This increase in the number of co-induced genes may reflect the delay of the transcriptomic response observed in the susceptible line Emir, which, at 5 and 9 dpi, partially matched the already ongoing early response of resistant lines. Interestingly, at 5 and 9 dpi, Wonga responded via repression of a large set of genes, as compared to 75A:258 or Emir. The number of genes repressed in 75A:258 stayed at a relatively low level until the end of the experiment. There was no single gene that was repressed simultaneously in all three lines at any time point (Figure 2). As in the transcriptome sequencing assay, the 75A:258 was sampled at five time points (1, 5, 9, 16 and 23 dpi), whereas Wonga and Emir were sampled at three time points (1, 5 and 9 dpi); overall, there were 11 line × time point experimental variants considered. Seventeen genes were significantly upregulated in resistant lines (75A:258 and Wonga) at all time points, including 6 genes that were significantly upregulated in all variants (representing both resistant and susceptible lines). There were 43 genes with a significantly altered expression at 1 and 5 dpi in both resistant lines. A susceptible line Emir revealed a distinct expression profile of this subset of genes because only 14 of 43 had a significantly changed expression at 1 dpi, 22 at 5 dpi, and all (43) at 9 dpi. Such an observation highlighted the delayed response of Emir to inoculation, hypothetically contributing to its susceptibility to *D. toxica*. Indeed, from the 67 genes that had significantly changed expression in 75A:258 and Wonga at 1 dpi, only 16 revealed such a pattern in Emir at the same time. However, this number was raised to 26 at 5 dpi and 60 at 9 dpi. Thus, nine days after inoculation, the resistance response of Emir finally matched the responses generated by 75A:258 and Wonga on the first day. This provided extra available time (about one week) for the fungus to colonize plant tissues in the susceptible cv. Emir.

Among the 3018 genes showing a significantly changed expression in response to inoculation, transmembrane (TM) domains were found in 179 genes, kinase domains in 157 genes, leucine-rich repeat (LRR) domains in 66 genes, coiled-coil (CC) domains in 19 genes, nucleotide-binding site (NBS) domains in 17 genes, and Toll/interleukin−1 receptor (TIR) domains in 7 genes (Supplementary Table S3). Four genes (*TanjilG_02534*, *TanjilG_03867*, *TanjilG_06162*, *TanjilG_22640*) were composed of CC-NBS-LRR, three genes (*TanjilG_06163*, *TanjilG_21020*, *TanjilG_27608*) carried NBS-LRR, and one gene (*TanjilG_13709*) had TIR-NBS-LRR.

### 2.4. Early Reaction of Resistant Lines Was Based on the Activation of the Defense Response and Oxylipin Biosynthesis Genes

Genes with an expression that was significantly altered in response to *D. toxica* inoculation were subjected to a Gene Ontology enrichment analysis. Representation of the terms was compared to the whole-genome annotation. The most frequently overrepresented biological process term was “defense response,” which appeared in all time × line combinations, except at 1 dpi in a susceptible Emir (Table 3).

This is an expected outcome that highlights an early and specific response of resistant lines. 75A:258 at 1 dpi also activated “oxidation-reduction process”, “oxylipin metabolic process” and “oxylipin biosynthetic process”. The two latter terms were also overrepresented at 1 dpi in Wonga. These processes are key components of a plant’s antifungal defense response (see Discussion). The early response of Wonga also included terms indicating global transcriptome and metabolome reprogramming, which was represented by several ontology terms addressing regulatory functions at all major molecular levels. These included “RNA biosynthetic process”; “regulation of RNA biosynthetic process”; “transcription, DNA-templated”; “regulation of transcription, DNA-templated”; “regulation of gene expression”; “regulation of biosynthetic process”; “regulation of cellular biosynthetic process”; “regulation of primary metabolic process”; “regulation of cellular metabolic process”. Such an observation highlighted that Wonga responded very early to *D. toxica* inoculation and this reaction was very complex. 75A:258 activated global transcriptome and metabolome reprogramming at 5 dpi, boosting the same processes as Wonga at 1 dpi. Key processes related to reactive oxygen species deployment, antibiotic metabolism, and drug catabolism were activated in 75A:258 as early as 5 dpi, whereas, in Wonga, these processes were targeted at 9 dpi. Contrary to 75A:258 and Wonga, Emir did not activate any typical defense response component at 1 dpi, and the only significantly enriched process was associated with xyloglucan metabolism, which was putatively related with cell wall reinforcement (see Discussion). Such an observation indicated the lack of early and specific recognition of the pathogen by the Emir cultivar. The first genes related to the defense response were activated in Emir at 5 dpi, whereas the full response, like those observed in Wonga and 75A:258 at 1 and 5 dpi, appeared in Emir at 9 dpi (Supplementary Table S4).

Taking into consideration the molecular functions of differentially expressed genes, the most frequently overrepresented terms were those related with chitin-triggered immunity (annotated as “chitin binding”), oxidative burst (including “heme binding,” “oxidoreductase activity,” and “peroxidase activity”), as well as transcriptome reprogramming (represented by “sequence-specific DNA binding,” “DNA-binding transcription factor activity,” “transcription regulator activity”) (Table 4). At 1 dpi, the highest number of overrepresented molecular function ontology terms was found in 75A:258 (11), followed by Wonga (6) and Emir (3). Such a difference between *L. angustifolius* genotypes was still observed at 5 dpi, accounting for 19, 13, and 3 overrepresented terms, respectively. Thus, the difference in the number and type of overrepresented ontology terms identified during the first 5 dpi matched the observed differences between genotypes regarding their resistance to *D. toxica*.

Weighted gene co-expression network analysis (WGCNA) revealed positive inoculation responsiveness of a group of genes controlling the “oxidation-reduction process”, “drug catabolic process” and “response to wounding” as well as genes controlling “gene expression” “ribosome biogenesis” and “ncRNA processing” (Supplementary Tables S5 and S6). Such an observation evidenced orchestrated transcriptomic control of major plant resistance mechanisms involving the oxidative burst, deactivation of toxic metabolites produced by the fungus, and protection of physically damaged tissues. WGCNA analysis also highlighted considerable differences between genotypes (irrespective of the applied inoculation), as visualized by the induced expression of genes in 26 modules and reduced expression of genes in 8 modules in the 75A:258 line compared to Emir. Wonga was found to have an intermediate gene expression pattern.

Taking into consideration the fact that there is a latent phase during disease development that gives some extra time for susceptible plants to react, we expected activation of the defense response in all studied lines. Therefore, we instead focused on the faster reaction of resistant lines than on the divergence of their response. Nevertheless, 269 genes were significantly upregulated only in 75A:258 and 273 genes with such a pattern in Wonga. These sets of genes differed by the composition of overrepresented GO terms in all three major annotation classes: biological processes (“cell redox homeostasis” and “oxidation-reduction process” vs. “pathogenesis” and “transmembrane transport”), cellular components (“cell surface” vs. “extracellular region”), and molecular functions (i.e., “oxidoreductase activity” vs. “transporter activity”). The list of overrepresented terms with statistical support is provided in Supplementary Table S7.

### 2.5. Genes from Defense Response Pathways Experienced up to a Thousandfold Upregulation in Inoculated Plants

Based on the results of differential gene expression profiling (Supplementary Table S8) and Gene Ontology enrichment analysis, 12 genes were selected for quantitative evaluation of their expression using real-time PCR. All these genes were found using RNA-seq survey to be highly upregulated by inoculation; however, the levels and timing of this upregulation differed between particular lines (Table 5).

**Figure 3:**
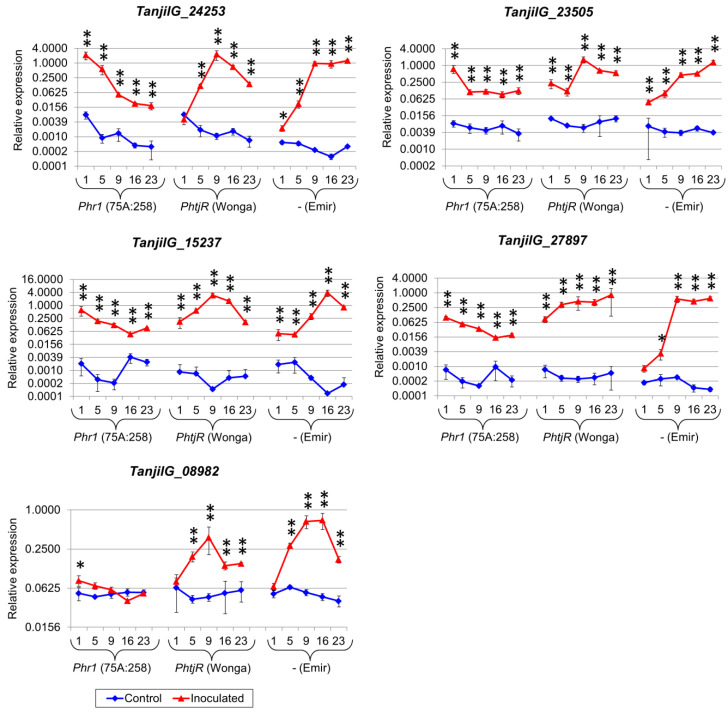
Expression profiles of genes that revealed the maximum level at the first time point in the 75A:258 line. The numbers 1, 5, 9, 16 and 23 stand for days post inoculation. *LanDExH7* and *LanTUB6* were used for the normalization, and *LanTUB6* dilution was used for inter-run calibration. The error bars indicate the standard deviation of three biological replicates, with each representing a mean of three technical replicates. The statistical significance of differences in the expression levels between the inoculated and control plants are marked above the data points (* *p*-value ≤ 0.05, ** *p*-value ≤ 0.001). A logarithmic scale was used to accommodate the observed differences in gene expression. Analyzed lines of *Lupinus angustifolius*: 75A:258 (highly resistant, carrying the *Phr1* gene), Wonga (moderately resistant, carrying the *PhtjR* gene), and Emir (susceptible).

**Figure 4:**
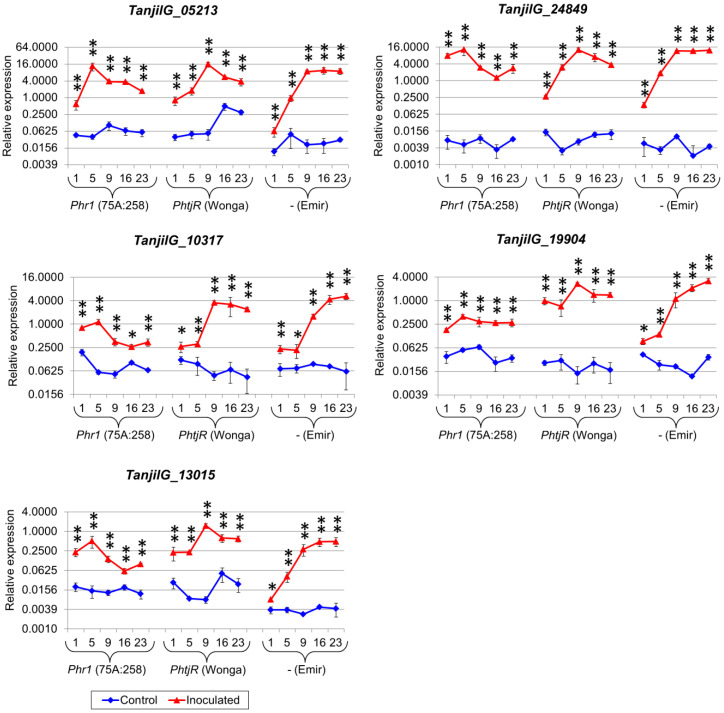
Expression profiles of genes that revealed the maximum level at the second time point in the 75A:258 line. The numbers 1, 5, 9, 16 and 23 stand for days post inoculation. *LanDExH7* and *LanTUB6* were used for the normalization, and *LanTUB6* dilution was used for inter-run calibration. The error bars indicate the standard deviation of three biological replicates, each representing a mean of three technical replicates. The statistical significance of differences in the expression levels between the inoculated and control plants are marked above data points (* *p*-value ≤ 0.05, ** *p*-value ≤ 0.001). A logarithmic scale was used to accommodate the observed differences in gene expression. Analyzed lines of *Lupinus angustifolius*: 75A:258 (highly resistant, carrying the *Phr1* gene), Wonga (moderately resistant, carrying the *PhtjR* gene), and Emir (susceptible).

**Figure 5:**
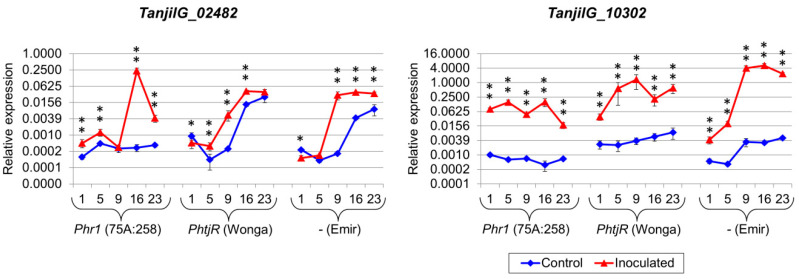
Expression profiles of genes that revealed the maximum level at the fourth time point in the 75A:258 line. The numbers 1, 5, 9, 16 and 23 stand for days post inoculation. *LanDExH7* and *LanTUB6* were used for the normalization, and *LanTUB6* dilution for the inter-run calibration. The error bars indicate the standard deviation of three biological replicates, each representing a mean of three technical replicates. The statistical significance of differences in the expression levels between the inoculated and control plants are marked above data points (* *p*-value ≤ 0.05, ** *p*-value ≤ 0.001). A logarithmic scale was used to accommodate the observed differences in gene expression. Analyzed lines of *Lupinus angustifolius*: 75A:258 (highly resistant, carrying the *Phr1* gene), Wonga (moderately resistant, carrying the *PhtjR* gene), and Emir (susceptible).

These genes included known components of plant defense responses, namely glutathione S-transferase-like genes (*TanjilG_24849* and *TanjilG_24253*), a basic endochitinase-like gene (*TanjilG_05213*), a putative lipid transfer protein gene (*TanjilG_08982*), WRKY transcription factor 75-like genes (*TanjilG_19904* and *TanjilG_10317*), a GDSL esterase/lipase 1-like gene (*TanjilG_13015*), a phospholipase A1-IIgamma-like gene (*TanjilG_27897*), a germin-like protein subfamily 1-like gene (*TanjilG_02482*), and a cyanogenic beta-glucosidase-like gene (*TanjilG_23505*). Two hypothetical genes without significant sequence similarity to other functionally annotated proteins (*TanjilG_15237* and *TanjilG_10302*) were also selected due to their extremely high responsiveness to inoculation in the RNA-seq experiment. Real-time PCR profiling confirmed the responsiveness of the selected genes to inoculation in all lines and highlighted significant differences in transcriptomic response between the genotypes (Table 6, Supplementary Table S9). Taking into consideration the timing of the upregulation, in the highly resistant 75A:258 line, five genes (*TanjilG_24253*, *TanjilG_08982*, *TanjilG_27897*, *TanjilG_23505*, and *TanjilG_15237*) revealed the highest expression level at 1 dpi (Figure 3), another five (*TanjilG_24849*, *TanjilG_05213*, *TanjilG_19904*, *TanjilG_10317*, and *TanjilG_13015*) at 5 dpi (Figure 4), and the remaining two at 16 dpi (Figure 5).

In the moderately resistant cultivar Wonga, no gene from this subset showed the highest expression at 1 or 5 dpi; the expression of ten genes peaked at 9 dpi, *TanjilG_02482* peaked at 16 dpi, and *TanjilG_27897* peaked at 23 dpi. In the susceptible cultivar Emir, no gene from this subset had the maximum expression at the first three time points; five genes (*TanjilG_05213*, *TanjilG_08982*, *TanjilG_02482*, *TanjilG_15237*, and *TanjilG_10302*) reached their maximum level at 16 dpi and seven genes at 23 dpi.

Genes with the highest mean upregulation after inoculation (averaged across all time points) in the 75A:258 line were as follows: *TanjilG_24849* (942-fold increase), *TanjilG_15237* (272-fold), *TanjilG_24253* (213-fold), *TanjilG_10302* (167-fold), *TanjilG_02482* (141-fold), *TanjilG_27897* (132-fold), *TanjilG_05213* (96-fold), and *TanjilG_23505* (37-fold). Most of these genes were also highly upregulated after inoculation in cultivar Wonga, including *TanjilG_15237* (5403-fold), *TanjilG_27897* (1069-fold), *TanjilG_24849* (757-fold), *TanjilG_24253* (565-fold), *TanjilG_10302* (139-fold), *TanjilG_23505* (87-fold), and *TanjilG_05213* (77-fold). Mean values of the upregulation after inoculation in cultivar Emir were even higher for some genes, namely, *TanjilG_15237* (10,029-fold), *TanjilG_24253* (2587-fold), *TanjilG_27897* (2068-fold), *TanjilG_24849* (2043-fold), *TanjilG_10302* (661-fold), *TanjilG_05213* (234-fold), and *TanjilG_23505* (117-fold). Interestingly, the revealed differences in mean upregulation values between genotypes did not follow the observed differences in resistance to *D. toxica*. However, in plant resistance to pathogens, the success of the host organism’s response is usually based on quick recognition of the infection rather than on the final amount of particular transcripts in a delayed response.

**Figure 6:**
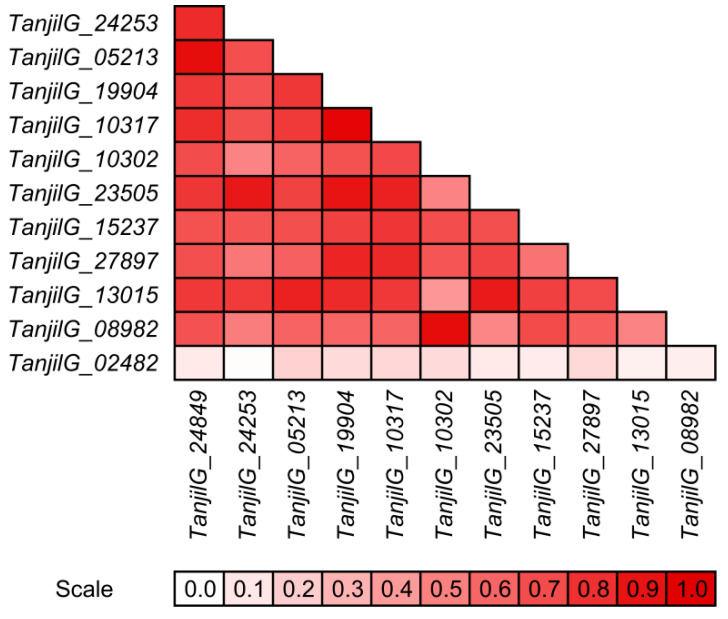
Correlations between gene expression profiles that were revealed using quantitative PCR.

Interestingly, some gene pairs revealed very similar expression profiles, both in the context of observed trends during the experiment, as well as revealed differences in expression levels between genotypes and variants (control and inoculated). Such similarities, visualized using high Pearson correlation coefficients, were identified for the following pairs: *TanjilG_10317* and *TanjilG_19904* (correlation of 0.95), *TanjilG_05213* and *TanjilG_24849* (0.92), *TanjilG_08982* and *TanjilG_10302* (0.92), *TanjilG_23505* and *TanjilG_24253* (0.89), *TanjilG_23505* and *TanjilG_19904* (0.89), *TanjilG_23505* and *TanjilG_13015* (0.88), *TanjilG_23505* and *TanjilG_10317* (0.85), *TanjilG_13015* and *TanjilG_05213* (0.85), *TanjilG_27897* and *TanjilG_19904* (0.84), and a few other combinations (Figure 6).

The high similarity of gene expression profiles may indicate their co-regulation by the same component(s). However, identification of this regulatory agent(s) would require further tests involving the silencing of candidate genes. Unfortunately, such studies are hampered in *L. angustifolius* due to the lack of an efficient transformation system that has been optimized for this species.

## 3. Discussion

### 3.1. Genotype Profiling of Diaporthe toxica Isolates

The analysis revealed that the genetic diversity of *D. toxica* isolates correlated with the host plant species (*L. luteus* vs. *L. angustifolius*) and not with geographic origin (Poland vs. Australia). However, it should be noted that the first Australian *L. luteus* variety, Wodjil, was released relatively recently and it was derived from a single plant selection originating from the Polish cultivar Teo, which was developed by Poznan Plant Breeders Ltd. in Poland [41]. Therefore, Polish and Australian *L. luteus* hosts should be considered genetically related.

The taxonomic identification of *Diaporthe/Phomopsis* has been traditionally based on host association because morphological characters are few and not reliable [42]. In the present study, RAPD-PCR and ITS markers were harnessed to genotype several *D. toxica* isolates and profile their molecular diversity. This approach facilitated the preselection of isolates for disease resistance experiments. Molecular-based diversity surveys are becoming a common approach in *Diaporthe*/*Phomopsis* studies. Among others, RAPD-PCR assays were used for profiling the *Diaporthe eres* complex in fruit trees (several species), *Diaporthe vaccinii* in cranberries (*Vaccinium macrocarpon*), and *Diaporthe phaseolorum* in soybean (*Glycine max*) [43,44,45]. ITS has been widely adopted for numerous studies, including those on *Diaporthe ambigua*, *Diaporthe angelicae*, *Diaporthe aspalathi*, *Diaporthe australafricana*, *D. eres*, *Diaporthe helianthi*, *Diaporthe infecunda*, *Diaporthe lusitanicae*, *Diaporthe neotheicola*, *Diaporthe perjuncta*, *D. phaseolorum*, *Diaporthe theicola*, *D. vaccinii*, *Diaporthe viticola*, and other *Diaporthe* species [43,44,46,47,48,49,50]. These studies highlighted the applicability of PCR-based methods for the evaluation of *Diaporthe* spp. diversity on numerous host plant species.

### 3.2. Mechanisms Involved in Lupinus angustifolius’ Defense Response to Diaporthe toxica

The results of gene expression profiling provided evidence supporting our hypothesis on the immunity reaction against *D. toxica* for 75A:258, and to some extent, for Wonga. These two resistant lines appeared to activate different responses to infection, with 75A:258 showing a spike in induced genes at 5 dpi, followed by a severe decrease, to rise again at 16 dpi, hitting a similar peak at 23 dpi; in contrast, Wonga revealed a continuously increasing trend that resembled a susceptible line (Table 2). The observed pattern for 75A:258 reflected the expected resistance response of a plant to the progress of the infection. The study on Phomopsis stem blight in *L. angustifolius* revealed that a few days after *D. toxica* infection, colonization of plant tissues slowed down and subcuticular coralloid hyphae were formed, indicating the beginning of the latent phase of the disease [22]. In that experiment, the number of coralloid structures observed in a susceptible cultivar Yandee was ≈185 times higher than in the resistant line 75A:258; moreover, these structures in Yandee were also ≈194 times larger than in 75A:258. Thus, a much lower subcuticular area was covered by the coralloid hyphae, reduced in the resistant 75A:258 line approximately 36,000-fold (as compared to the susceptible line) [22], would result in much lower stimulation of a defense response during the latent phase, causing this sudden drop in the number of induced genes after the initial boost. In field conditions, the latent phase lasts until plant maturity [20,21,28,29]. However, in a greenhouse, with the high humidity favoring fungal development, full disease symptoms are observed on susceptible plants no later than 30 dpi, indicating a considerable reduction of the latent phase [30]. Termination of the latent phase, which is expected to occur in a greenhouse about 15–20 dpi, would explain this sudden surge in defense response, resulting in the final arrest of the pathogen. Indeed, taking into consideration GO terms, from 48 terms that were overrepresented in the 75A:258 at 5 dpi and 38 at 23 dpi, 31 GO terms (65–82%) were overlapping. From 672 and 691 genes that were upregulated at 5 and 23 dpi, 360 (52–54%) were the same.

The induction and repression patterns observed in Wonga (Table 2) seemed to be similar to the susceptible line, although with increased sensitivity. Such an observation is in line with the results of disease phenotyping studies showing the development of some disease symptoms in Wonga and the high immunity of 75A:258 [22,26,29,30,51]. Nevertheless, considering the overrepresented GO terms, Wonga also showed a sharp drop in its defense response, observed at 5 dpi, followed by high induction at 9 dpi. Moreover, GO terms overrepresented in Wonga at 1 and 9 dpi highly overlapped with those in 75A:258 at 5 and 23 dpi. An increasing trend in the total number of genes upregulated in Wonga, along with the observed sudden decrease in overrepresented GO terms, may indicate that Wonga did not monitor the growth of *D. toxica* as precisely as 75A:258 did; however, it was enough to develop a successful defense. The number of uniquely upregulated genes was very similar in 75A:258 and Wonga; however, overrepresented terms did not overlap, indicating some divergence in their responses.

#### 3.2.1. Peroxidases and Reaction Oxygen Species

Three major gene ontologies associated with reactive oxygen species and overrepresented in inoculated plants were: GO:0042743 “hydrogen peroxide metabolic process,” GO:0072593 “reactive oxygen species metabolic process,” and GO:0042744 “hydrogen peroxide catabolic process.” This set of genes included 24 homologs of peroxidases, superoxide dismutase, and one hypothetical protein. Peroxidases were also major upregulated genes in a resistant wild relative of asparagus, *Asparagus kiusianus,* 24 h post inoculation with *Phomopsis asparagi*, which causes stem blight disease [52]. Peroxidases are well-known components of the plant resistance response and are induced in host tissues after an infection. They may slow down the infection propagation via cell wall reinforcements, especially via lignification, suberization, and cross-linking of particular compounds [53]. Lignification is considered as one of the mechanisms of plant resistance constituting a physical barrier that hampers the growth of the pathogen [54,55,56]. The induction of lignification-associated genes was also observed after inoculation with the hemibiotrophic fungus *Diaporthe ampelina* in a resistant hybrid grapevine [57]. Here, the upregulation of the lignin-forming anionic peroxidase-like (*TanjilG_03329*) and peroxidases A2-like (*TanjilG_27107*, *TanjilG_27110*, and *TanjilG_27109*) genes may be considered as a part of this process [58]. Another function of peroxidases is related to the oxidative burst, which is an early response of plants to biotic stress that is based on the production of huge amounts of reactive oxygen species [59]. Reactive oxygen species have many functions, including a hypersensitive response, the aforementioned strengthening of cell walls surrounding the infection site, and mediation of defense gene activation [60]. Here, a group of cationic peroxidase 1 homologs (*TanjilG_19270*, *TanjilG_21432*, *TanjilG_32594*, and *TanjilG_32595*) was found to be responsive to inoculation. The induction of cationic peroxidases is related to increased resistance to necrotrophic fungi, as well as rapid removal of hydrogen peroxide and improved lignin formation [61,62]. Besides peroxidases, superoxide dismutase (*TanjilG_09550*) was found to be upregulated. Superoxide dismutases are involved in the metabolism of reactive oxygen species and cooperate with catalases to control the appropriate level of hydrogen peroxide during infection [63,64].

#### 3.2.2. Glutathione S-Transferase-Like Genes

Two glutathione S-transferase-like (GST-like) genes (*TanjilG_24849* and *TanjilG_24253*) were found to be highly responsive to *D. toxica* inoculation in this study. The very resistant 75A:258 line revealed massive upregulation of GST as early as at the first time point (1 dpi), whereas other lines reached a comparable level of expression 4–8 days later. GSTs are known components of plant–pathogen interactions and their roles are attributed to the detoxification of toxic substances produced by the pathogens, attenuation of oxidative stress resulting from the plant’s defense response, and participation in hormone transport [65]. GSTs were revealed to be responsive to infections by a wide range of pathogens, including biotrophic, hemibiotrophic, and necrotrophic fungi. The examples of biotrophic plant–pathogen interactions include wheat (*Triticum aestivum*) and wheat powdery mildew (*B. graminis* f. sp. *tritici*) [66] and wild tomato (*Solanum habrochiates*) and tomato powdery mildew (*Oidium neolycopersici*) [67], and soybean (*Glycine max*) or wooly glicyne (*Glycine tomentella*) and rust (*Phakopsora pachyrhizi*) [68,69]. Antifungal GSTs’ role in hemibiotrophic interactions was evidenced for potato (*Solanum tuberosum* L.) and late blight (*Phytophthora infestans*) [70], barley (*Hordeum vulgare* L.) and *Fusarium graminearum* [71], *Nicotiana benthamiana* and *Colletotrichum* spp. [72], sorghum and *C. sublineolum* [73], *Lilium regale* and *Fusarium oxysporum* [74], *Arabidopsis* and several fungal pathogens [75], and wheat and *Fusarium* head blight [76]. Among the necrotrophic fungi, the most remarkable interactions with GSTs’ involvement include *Arabidopsis thaliana* or *Vitis* spp. and *Botrytis cinerea* [77,78,79], *A. thaliana* and *Altenaria brassiciola* [80,81,82], and oilseed rape (*Brassica napus*) and *Sclerotinia sclerotiorum* [83,84,85,86].

#### 3.2.3. WRKY Transcription Factors

Several WRKY transcription factors were revealed in this study to be very responsive to *D. toxica* inoculation. Expression profiles of two WRKY75-like genes (*TanjilG_19904* and *TanjilG_10317*) were evidenced to be highly correlated to each other, as well as to several other genes that were highly upregulated in response to inoculation (Figure 6). WRKY transcription factors constitute a large network of genes that are responsive to biotic and abiotic stresses, as well as regulating plant growth and development [87,88]. A large part of WRKYs is involved in transcriptome reprogramming associated with plant immune responses [89]. In this context, WRKYs are components of both pathogen-associated molecular-pattern-triggered immunity and effector-triggered immunity [40]. WRKY factors regulate the defense response at various levels, modulating the expression of target genes directly or indirectly by activating or repressing transcription factors, including other WRKY factors, as well as by interacting with chromatin-remodeling factors [90]. Numerous WRKYs were evidenced to participate in interactions between plants and pathogenic fungi. Interestingly, in such interactions, WRKYs were frequently revealed to regulate the expression of GST genes [65].

#### 3.2.4. Isoflavonoid Biosynthesis Pathway

Isoflavonoids function as antimicrobial phytoalexins and are upregulated in response to fungal infection or exposure to elicitors isolated from the cell walls of yeast or plant pathogenic fungi [91,92]. Antifungal compounds originating from the isoflavonoid pathway are also synthesized in lupins, including *L. angustifolius* and *L. albus* [93,94]. Isoflavone synthase (IFS) is the key enzyme that is responsible for isoflavonoid biosynthesis in plants [95]. *L. angustifolius’* genome contains three full-length genes that encode this enzyme [96]. One of these genes, *TanjilG_05543*, was found to be upregulated in response to *D. toxica* inoculation in this study. RNAi silencing of IFS genes in *Glycine max* resulted in enhanced susceptibility to a hemibiotrophic *Phytophthora sojae*, affecting both R gene-mediated resistance and horizontal resistance [97]. Other components of the isoflavonoid pathway upregulated in *L. angustifolius* after inoculation were isoflavone 2’-hydroxylase-like genes (*TanjilG_20118*, *TanjilG_21406*, and *TanjilG_16385*), isoflavone-7-O-methyltransferase 9-like genes (*TanjilG_10939* and *TanjilG_02547*), and an isoflavone 4’-O-methyltransferase-like (*TanjilG_05544*) gene. Isoflavone 2’-hydroxylase was revealed to be induced in chickpea (*Cicer arietinum* L.) cultivars that are resistant to a necrotrophic ascomycete fungus, *Ascochyta rabiei*, resulting in high accumulation of two pterocarpan phytoalexins, namely, medicarpin and maackiain [98,99]. Isoflavone-7-O-methyltransferase activity was found to be highly induced in *Medicago sativa* cell suspension cultures treated with elicitors originating from cell walls of the hemibiotrophic fungus *Colletotrichum lindemuthianum*, as well as from the yeast [100]. Moreover, overexpression of isoflavone-7-O-methyltransferase in *M. sativa* resulted in increased induction of phenylpropanoid/isoflavonoid pathway gene transcripts after infection and provided resistance to *Phoma medicaginis*, the *necrotrophic* fungal pathogen of alfalfa [101].

#### 3.2.5. Lipoxygenase Pathway

Oxylipins are oxidized products that are obtained from the metabolism of α-linolenic acids (18:3) or linoleic acid (18:2) released from chloroplast membranes [102]. Oxylipins play fundamental roles in plant defense [103,104,105]. The first step in this metabolic pathway is committed by lipoxygenases: (9S)-lipoxygenase, which produces (9S)-hydroperoxyoctadecatrienoic acid, or by (13S)-lipoxygenase, which produces (13S)-hydroperoxyoctadecatrienoic acid [102]. This study provided transcriptomic evidence for the involvement of five (9S)-lipoxygenase genes (*TanjilG_14647*, *TanjilG_15668*, *TanjilG_26769*, *TanjilG_31614*, and *TanjilG_18117*) in the *L. angustifolius* defense response to *D. toxica*. Interestingly, the expression levels of two lipoxygenase genes were also induced in *G. max* plants subjected to inoculation by *D. caulivora*, which is a pathogenic fungus that causes soybean stem canker [106]. Numerous studies demonstrated the involvement of the (9S)-lipoxygenase pathway in modulating oxidative stress, lipid peroxidation, and plant defense [107]. The (9S)-lipoxygenase pathway was revealed to control the modification of the cell wall (formation of polysaccharide deposits, which are composed of callose and pectin), as well as the production of reactive oxygen species [108]. The (9S)-lipoxygenase gene from cotton (*Gossypium hirsutum*) was associated with the hypersensitive reaction to bacterial (*Xanthomonas campestris*) infection involving programmed cell death [109]. Indeed, the transient expression of pepper (*Capsicum annuum*) (9S)-lipoxygenase promoted a cell death phenotype and defense responses [110]. The same study showed that overexpression of this gene in *Arabidopsis* enhances resistance to microbial pathogens (bacteria and fungi). Derivatives from the (9S)-lipoxygenase pathway, called maize death acids, were shown to perform direct phytoalexin activity against pathogens, mediate defense gene expression, and confer cytotoxicity resulting in cell death [111]. In this study, the (9S)-lipoxygenase pathway represented by gene ontologies GO:0031407 “oxylipin metabolic process” and GO:0031408 “oxylipin biosynthetic process” was upregulated only at the first time point in both resistant accessions. This observation is in line with reports on the negative correlation between induced systemic resistance and (9S)-lipoxygenase activity, as provided for a maize model [112,113,114].

#### 3.2.6. Xyloglucan Endotransglucosylases/Hydrolases

The only biological process that was overrepresented in a susceptible Emir at 1 dpi was GO:0010411 “xyloglucan metabolic process”, which was conferred by xyloglucan endotransglucosylase/hydrolase (*XTH*) genes (*TanjilG_09455*, *TanjilG_26141*, *TanjilG_26142*, and *TanjilG_26144*). Xyloglucans are molecules that cross-link cellulose microfibrils and form a xyloglucan-cellulose network, which provides cell wall structural strengthening [115,116]. The plant cell wall constitutes the first external barrier to invading pathogens. Two types of enzymes performing xyloglucan modifications have been identified hitherto, namely, xyloglucan-specific endo-hydrolases and xyloglucan endotransglucosylases, which are collectively designated as xyloglucan endotransglucosylases/hydrolases (XTHs). XTHs are involved in numerous developmental processes, including plant growth, fruit ripening, seed mobilization, root aerenchyma formation, abscission zone development, and vascular tissue establishment [117,118]. *XTH* genes are responsive to various abiotic stress conditions, including drought and salinity [119,120,121]. Moreover, *XTH* genes contribute to plant resistance against bacteria, fungi, and pests. Indeed, transcriptional control of plant *XTH*s expression by the fungus, causing fruit softening and wall disassembly, was proposed as a putative process that facilitates the colonization of tomato fruit by *Penicillium expansum* [122]. *XTH* gene expression was also downregulated during the early phase of tomato root interaction with plant-growth-promoting rhizobacteria, enabling partial cell wall disassembly and endorhizosphere colonization [123]. In contrast, during the setup of an arbuscular mycorrhizal symbiosis between *Medicago truncatula* and *Glomus versiforme*, the induction of an *XTH* gene was observed [124]. *XTH* genes are involved in plant defense responses to inoculation by pathogenic fungi, as evidenced by interactions between *Arabidopsis* and the necrotrophic *Rhizoctonia solani* (the causal agent of root rot disease), between wild jute species and the *hemibiotrophic* *Macrophomina phaseolina* (causing stem rot disease), and between sugarcane and a biotrophic *Sporisorium scitamineum* (responsible for culmicolous disease) [125,126,127]. *XTH* genes were also revealed to be involved in the phloem response to aphid infestation of celery (*Apium graveolens*) and *Arabidopsis* [128,129].

#### 3.2.7. Systemic Acquired Resistance Signaling

One of the six genes that were significantly upregulated in response to *D. toxica* inoculation for all studied lines and time points was a homolog of an *A. thaliana* gene encoding defective in induced resistance 1 (DIR1) protein. DIR1 belongs to non-specific lipid transfer proteins and is required for the generation and propagation of systemic acquired resistance (SAR) [130,131,132,133]. When SAR is induced, DIR1 protein is transported via phloem from the infection site to distant organs, serving as a mobile SAR signal [134,135,136,137]. DIR1 contributes to conferring SAR induction by using several signaling compounds, including azelaic acid, dehydroabietinal, glycerol-3-phosphate, and methyl salicylate (MeSA) [138,139,140,141]. DIR1 orthologs were found in several plant families represented by tobacco, tomato, cucumber, and soybean, providing the suggestion that DIR1-mediated SAR signaling might be conserved [142]. Our study provided novel evidence for positive, rapid, and long-lasting DIR1 transcriptional responsiveness to pathogenic fungus infection, indicating its involvement in SAR development and maintenance in *L. angustifolius*.

#### 3.2.8. Pathogenesis-Related Protein Class PR10

The overrepresented Gene Ontology term GO:0006952 “defense response” concerned, among others, the LlR18B (*TanjilG_26536*) and LlR18A (*TanjilG_27014* and *TanjilG_27015*) proteins. These sequences belong to the PR10.1 class of pathogenesis-related proteins [143]. LlR18A was constitutively expressed in roots but accumulated in leaves in response to pathogenic bacteria [144]. It belongs to the same group as rice RSOsPR10 protein, which is rapidly induced by a fungal infection, possibly through activation of the jasmonic acid signaling pathway [145]. Expression of *L. luteus* PR10.1 protein genes was primarily localized in roots, whereas genes encoding PR10.2 proteins were active in all organs and showed responsiveness to wounding, oxidative stress, and salicylic acid treatment [144,146]. Other PR10 genes that significantly activated in response to *D. toxica* in this study included 11 homologs of stress-induced protein starvation-associated message 22 (SAM22), which is located in the cluster of tandem repeats neighboring the LlR18B protein. The whole cluster revealed a similar pattern of transcriptional profiles. SAM22 mRNA was revealed to accumulate in cultured soybean cells (*Glycine max*) in response to cytokinin or auxin starvation [147]. Interestingly, SAM22 was also highly responsive (30–50-fold increase) in leaves exposed to salicylic acid, methyl viologen (i.e., paraquat), chitosan (a fungal elicitor), and hydrogen peroxide [148]. These reports, along with the observations from the present study, support the hypothesis of the putative involvement of *L. angustifolius* LlR18A, LlR18B, and SAM22 homologs in the defense response against *D. toxica*.

#### 3.2.9. Concluding Remarks

Based on the results of the transcriptomic profiling, the conclusion could be drawn stating that the resistance of *L. angustifolius* to *D. toxica* was based on the early activation of a defense response. Thus, the *L. angustifolius* line susceptible to *D. toxica* (Emir) developed a full defense response about 4–8 days later than the resistant germplasm carrying *Phr1* or *PhtjR* genes. The early defense response of *L. angustifolius* germplasm that was resistant to *D. toxica* was associated with the induction of genes controlling reactive oxygen species deployment, oxylipin biosynthesis, and global transcriptome reprogramming. Furthermore, the defense response of *L. angustifolius* to *D. toxica* was related to a high upregulation of genes encoding glutathione S-transferases, WRKY transcription factors, pathogenesis-related proteins, and enzymes from isoflavonoid biosynthesis pathway, as well as typical components of systemic acquired resistance, such as the DIR1 protein. Numerous genes remained upregulated until the end of the experiment (23 days), indicating a long-lasting defense response that successfully prevented pathogen growth in the latent phase of the infection.

## 4. Materials and Methods

### 4.1. DNA Polymorphisms of D. toxica Isolates

A collection of ten isolates of *D. toxica* [30] was analyzed in the study. DNA was extracted according to the method described by Irzykowski [149]. Briefly, 7-day-old mycelia of the selected isolates of *D. toxica*, grown in shaking liquid Czapek-Dox medium (Sigma-Aldrich, Merck Group, St. Louis, MO, USA) supplemented with yeast extract 2 g/L (Sigma-Aldrich, Merck Group, St. Louis, MO, USA), were separated from the medium using the vacuum pump PL 2-2 (AGA LABOR, Warsaw, Poland) and freeze-dried (Heto Lab Equipment, Allerød, Denmark). DNA extraction was done using the DNeasy Plant Mini Kit (Qiagen, Hilden, Germany). Random Amplified Polymorphic DNA (RAPD) analyses were carried out with four randomly chosen primer sets: OPC, OPG, OPJ, and OPL (Operon Technologies, Alameda, CA, USA). The primers (*n* = 80) were initially screened and 6 primers, showing unambiguous polymorphic bands, were chosen (Supplementary Table S1).

RAPD amplifications were carried out under mineral oil, in 10 μL final volumes containing 1 μL of template DNA, 200 μM of dNTP, 1 μM of primer, 0.5 U of Taq polymerase (Qiagen) in 1× PCR buffer (Qiagen). The buffer was supplemented with a solution of 3 mM MgCl_2_. Amplifications were performed in a C1000 thermal cycler (Bio-Rad, Hercules, CA, USA). The PCR program was composed of the following steps: (1) initial DNA denaturation for 2 min at 94 °C; (2) 45 cycles of (2a) 30 s at 94 °C, (2b) 1 min at 36 °C, (2c) 2 min at 72 °C; (3) final extension at 72 °C for 5 min. The RAPD-PCR products were stored at 4 °C. The separation of fragments was done on a 2% agarose gel (Life Technologies, Carlsbad, CA, USA) in a 1× TBE buffer, stained with ethidium bromide. PCR product visualization was done by Scion Image Beta 3b (Scion Corporation, Frederick, MD, USA). The PCR products were scored and analyzed using TREECON for Windows version 1.3b, University of Antwerp, Wilrijk–Antwerpen, Belgium [150].

The analysis of polymorphisms in the ITS1-5.8S-ITS2 fragment was done using the forward primer WIRZ G1 [149] and reverse primer PN10 [151]. Amplifications were carried out in a 4.5 μL final volume containing 0.3 μL of template DNA, 200 μM of dNTP, 2 μM of each primer, 0.5 U of Taq polymerase (Qiagen), and 3 mM MgCl_2_ in a 1 × PCR buffer (Qiagen). The reaction was performed in a C1000 thermal cycler (Bio-Rad) using the following program: (1) denaturation at 94 °C for 2 min; (2) 45 cycles of (2a) 30 s at 94 °C, (2b) 30 s at 60 °C, (2c) 1 min at 72 °C; (3) final extension at 72 °C for 5 min. PCR products were cleaned from the dNTPs and primers using 0.3 μL FastAP thermosensitive alkaline phosphatase and exonuclease I (Thermo Fisher Scientific, Waltham, MA, USA). Sequencing of the ITS fragments was done using an ABI Prism 310 Genetic Analyzer, an ABI PRISM BigDye V3.0 Terminator Cycle Sequencing Ready Reaction Kit, and AmpliTaq DNA Polymerase (Perkin Elmer, Waltham, MA, USA), according to the methods described by the manufacturer. DNA sequences were inspected using Chromas 1.43 (Technelysium, South Brisbane, QLD, Australia). The sequences were compared and aligned using Clustal X ver. 1.81, (UCD Conway Institute, University College Dublin, Dublin, Ireland) [152]. Sequences were checked for species identification confirmation using the GeneBank and the EMBL Nucleic Acid Database, with the BLAST algorithm [153].

### 4.2. Plant Material

Seven lines were assessed in the study: 75A:258, Wonga, Emir, Tanjil, Unicrop, and Baron. 75A:258 is an Australian *L. angustifolius* breeding line that was derived from a moderately resistant wild line from Morocco, namely, CPI65211A, and a cross-derivative of cultivars Marri and P22872 [23]. This line carries the resistant *Phr1* allele and is highly resistant to *D. toxica*, as evidenced by independent studies in Australia and Poland [26,28,29,30]. Wonga is an Australian *L. angustifolius* cultivar that was released in 1996 and was obtained as a cross between the F7-derived selection from Gungurru and a P22721 line (wild type from Spain) [154]. Wonga carries the resistant *PhtjR* allele [25]. Its resistance to *D. toxica* has been evidenced across many experimental sites since the early 1990s [25,30,51,154]. Emir is a Polish early flowering *L. angustifolius* cultivar that was created in 1981 in the experimental station Przebędowo of Plant Breeding Smolice Ltd. [155]. This cultivar does not have resistant *Phr1* and *PhtjR* alleles and was found to be highly susceptible to *D. toxica* in both our previous experiments conducted in 2007 and 2017 [30]. For reference, several other lines were included in the disease resistance assay, namely Tanjil (1998 Australian cultivar derived as single plant selection form Wonga, resistant to *D. toxica*), Unicrop (1971 Australian cultivar carrying early flowering gene, susceptible to *D. toxica*) and Baron (2002 Polish cultivar). Seeds of 75A:258 and Wonga were shared by Hua’an Yang from the Department of Agriculture and Food Western Australia in 2003, whereas Emir, Baron, Tanjil, and Unicrop were provided by Plant Breeding Smolice Ltd. (breeding station Przebędowo).

### 4.3. Genotyping for the Phr1 and PhtjR Alleles

Lines were validated prior to the experiments for the presence of desired alleles using Ph258M1 and Ph258M2_dCAPS markers for the *Phr1* gene, and PhtjM7_dCAPS2, InDel2, and InDel10 markers for *PhtjR* [25,26,27,30,51]. DNA was isolated from 2-week-old leaves (two biological replicates) using a DNeasy Plant Mini Kit (Qiagen) without any changes to the protocol. The quality and concentration of isolated DNA were evaluated using agarose gel electrophoresis with an SYBR Safe DNA Gel Stain (Thermo Scientific) UV-light visualization and spectrophotometer measurements (NanoDrop 2000; Thermo Scientific). The primers, PCR conditions, restriction enzymes, and agarose gel electrophoresis were provided in our recent paper on the validation of *D. toxica* resistance markers [30].

### 4.4. D. toxica Experiment in Controlled Conditions

The isolate that showed the highest virulence against *L. angustifolius* in our previous study (DTOXA2, Perth, WA, Australia, or WAC8782, Wagga Wagga, NSW, Australia) was selected [30]. The experiment was performed in a controlled environment (a computer-controlled greenhouse) with a temperature regime of a 21 °C day/16 °C night and under a 14 h photoperiod. Pots (11 × 11 × 21 cm) with sterilized soil (TS-1 REC 085 Medium Basic, Klasmann-Dellman Polska, Warsaw) were used. The inoculation was performed 4 weeks after sowing at 10 a.m. The lower parts of stems were delicately scarified using a lancet (5 cm from root neck) and inoculated with 20 μL of conidia suspension of a given isolate of *D. toxica* (10^6^ conidia per mL). The inoculation sites were wrapped with Parafilm (Sigma-Aldrich) to minimize evaporation. Control plants were also scarified but no inoculum was applied. After the inoculation, the plants were grown in relative humidity above 80%. Tissue sampling was performed at 10 a.m. at 1, 5, 9, 16 and 23 dpi. Four biological replicates per each time point, line, and variant were sampled, immediately frozen in liquid nitrogen, and stored at −80 °C until the RNA extraction. Every sample consisted of a 10 mm stem section carrying the scarification site (in the middle) with the surrounding regions, weighing approximately 90 mg. The scoring of the Phomopsis stem blight symptoms [30] was performed on the remaining plants at 30 dpi. The disease scoring was done using a scale from 1 (immune, no symptoms) to 9 (fully susceptible).

### 4.5. RNA Isolation

Every frozen plant sample was divided into two parts (45 mg each) and processed in parallel. Stem fragments were cut into five pieces (about 10 mg each) and homogenized using a TissueLyser II (Qiagen) and two stainless steel beads Ø5 mm (Qiagen) in 2 mL tubes (Eppendorf, Hamburg, Germany). RNA isolation was performed using a Spectrum Plant Total RNA Kit (Sigma-Aldrich) with some alterations to the standard lysis protocol based on our previous experiences. Namely, 20 µL of 2-mercaptoethanol (instead of the proposed 10 µL) was added for every 1 mL of lysis solution. Moreover, 650 µL of the lysis solution (instead of the proposed 500 µL) was added to 45 mg of tissue powder (instead of the proposed 100 mg). The incubation was performed for 3 min at 56 °C, followed by centrifugation for 5 min to pellet the cellular debris. A total of 370 µL of lysate supernatant was retained each time (amount not provided in the protocol). Centrifugation during the lysate filtration step was performed at maximum speed for 2 min (instead of the proposed 1 min). A total of 750 µL of the binding solution (instead of the proposed 500 µL) was pipetted into the cleared lysate. The on-column DNase digestion was also modified by providing an additional centrifuge step (90 s at maximum speed) to dry the column before the application of the DNase I mixture. Moreover, the DNase I digestion was prolonged to 25 min (instead of the proposed 15 min). The other steps were identical to the protocol. The RNA purity and integrity were measured using an Experion™ Automated Electrophoresis System (Bio-Rad). The RNA concentration, A260/A280 ratio, and RNA quality indicator (RQI) of the samples prepared for sequencing are provided in Supplementary Table S2. The RNA concentration and A260/A280 ratio of samples prepared for the quantitative PCR profiling are provided in Supplementary Table S10.

### 4.6. RNA Sequencing and Data Analysis

Three biological replications were analyzed for each experimental variant. For the 75A:258 line, samples collected at all five time points were used, whereas, for the Wonga and Emir cultivars, samples from the first three time points were used. RNA libraries were prepared using a TruSeq RNA Sample Prep Kit v2 and sequenced on an Illumina 6000 platform using the 100 bp paired-end protocol (Macrogen Europe, Amsterdam, Netherlands). After removing the adapter-related sequences and quality trimming using AdapterRemoval ver 2.1.7 [156] (parameters:-minquality 20-minlength 50), the data were mapped in the reference sequence of *L. angustifolius* LupAngTanjil_v1.0 (EnsemblPlants) using TopHat ver. 2.1.1 [157] (parameters:-no-mixed-library-type fr-unstranded-no-discordant). Reads that were aligned to annotated transcripts were counted using the function featureCounts in Bioconductor, R 3.5.1 (Rsubread library [158]), and the count data were submitted for differential expression analysis in Deseq2 [159]. Differentially expressed genes in defined comparisons were declared as those that were characterized by a base mean expression of at least 5, |log_2_(Fold Change)| > 2, and corrected *p*-value < 0.05. A Gene Ontology terms enrichment analysis was performed using the hypergeometric test, with computation of the family-wise error rates (FWER), using the GOfuncR library in Bioconductor [160]. A weighted gene co-expression network analysis was performed using the WGCNA library in R [161,162] (parameters: beta = 6, average link clustering method, cutHeight = 0.60, minSize = 40). Genes that significantly altered the expression in response to inoculation were screened for the presence of typical domains using the Disease Resistance Analysis and Gene Orthology (DRAGO 2) tool in the Plant Resistance Genes database (PRGdb) [163].

### 4.7. Selection of Genes for Quantitative Expression Profiling

Reference genes reported in previous *L. angustifolius* quantitative gene expression studies were selected for this experiment, namely *LanTUB6* (Lup032899, XM_019581544.1) and *LanDExH7* (Lup023733, XM_019579367.1) [164,165,166]. Names provided in the parentheses correspond to the gene loci from the *L. angustifolius* genome [38] and to LupAngTanjil_v1.0 NCBI Reference Sequences, respectively. The selected genes for quantitative expression profiling were based on the results of differential RNA-seq analysis and literature data. In general, genes having the highest number of time points and genotype samples with significantly altered expressions and the highest log_2_-fold changes in response to inoculation were selected for the PCR-based quantification. This set included Lup024849 (*TanjilG_24849*, XM_019588232.1), Lup002313 (*TanjilG_02313*, XM_019569456.1), Lup023505 (*TanjilG_23505*, XM_019598586.1), Lup005213 (*TanjilG_05213*, XM_019597770.1), Lup019904 (*TanjilG_19904*, XM_019580465.1), Lup013015 (*TanjilG_13015*, XM_019601877.1), Lup010317 (*TanjilG_10317*, XM_019574134.1), Lup002482 (*TanjilG_02482*, XM_019577320.1), Lup027897 (*TanjilG_27897*, XM_019590187.1), Lup024253 (*TanjilG_24253*, XM_019608787.1), Lup010302 (*TanjilG_10302*), Lup015237 (*TanjilG_15237*, XM_019604835.1), and Lup008982 (*TanjilG_08982*, XM_019569344.1). The primers were designed in Geneious Prime (Biomatters Ltd., Auckland, New Zealand) using Primer3 [167,168] while targeting PCR product range 150–250 bp. The designed primers and expected PCR product sizes are provided in the Supplementary Table S11.

### 4.8. Quantitative Gene Expression Analysis

A CFX Connect Real-Time PCR Detection System (Bio-Rad) and 96-well PCR plates (Bio-Rad) were used for quantitative profiling. First, the instrument was calibrated using a Melt Calibration Kit (Bio-Rad) according to the protocol. Then, standard curves were developed for each analyzed gene following recent recommendations [169]. PCR products were amplified using GoTaq G2 Flexi DNA Polymerase (Promega, Madison, WI, USA) and proceeded to 1% agarose gel electrophoresis. Amplicons were excised from a gel, extracted using QIAquick Gel Extraction Kit (Qiagen), quantified using NanoDrop 2000 (Thermo Fisher Scientific), and directly sequenced (Genomed Ltd., Warsaw, Poland). Dilution series ranging from 1 to 10^−9^ times the original template concentrations were prepared using an initial volume of 20 µL to reduce pipetting errors. Three technical replicates per each concentration were performed using iTaq Universal SYBR Green Supermix (Bio-Rad). A two-step PCR protocol was applied. *R*^2^ and PCR efficiency values were calculated in CFX Manager 3.1 (Bio-Rad). Calculated values are provided in Supplementary Table S12.

First-strand cDNA synthesis was performed using an iScript cDNA synthesis kit (Bio-Rad) and 4 μg of total RNA per sample. An inter-run calibration sample (*LanTUB6*) and no template control were used on all plates. All PCR runs were performed using three technical repeats. To monitor the specificity of the amplification, high-resolution DNA melting in the temperature range from 65 to 85 °C was performed after each PCR. Unspecific products (or primer dimers) were detected as additional melting peaks at different temperatures than those obtained during the standard curve preparation. Calculations of ∆∆Cq were performed in CFX Manager 3.1 (Bio-Rad) by taking into consideration the PCR quantification results obtained for both reference genes. Final computations and visualizations (graphs) were done using Microsoft Excel 2010 (Microsoft, Redmont, WA, USA).

## Figures and Tables

**Table 1:** Comparison of the *Phr1* (Ph258M1 and Ph258M2) and *PhtjR* (PhtjM7, InDel2, and InDel10) marker polymorphisms and resistances to *D. toxica*.

Accession	Line	Ph25M1 *Phr1*	Ph258M2 *Phr1*	PhtjM7 *PhtjR*	InDel2 *PhtjR*	InDel10 *PhtjR*	Disease Index 2017 ^1^	Disease Index 2018 ^2^	Resistance Genes
96191	Wonga	S ^3^	S	R	R	R	1.8 ± 1.3	1.3 ± 0.4	*PhtjR*
96214	Tanjil	S	S	R	R	R	2.2 ± 1.4	2.2 ± 1.4	*PhtjR*
26979	75A:258	R ^4^	R	S	S	S	1.7 ± 1.3	1.7 ± 1.3	*Phr1*
96102	Unicrop	S	S	S	S	S	3.4 ± 1.7	3.4 ± 1.5	-
96121	Emir	S	S	S	S	S	5.2 ± 1.8	4.1 ± 1.1	-
96210	Baron	S	S	S	S	S	4.3 ± 1.3	5.2 ± 1.4	-

^1^ Data from a recently published experiment [30]. ^2^ Data from an experiment performed for gene expression profiling (this study). ^3^ S—susceptible allele. ^4^ R—resistant allele.

**Table 2:** Number of genes with significantly altered expression in response to *Diaporthe toxica* inoculation.

Line	Response	1 dpi ^1^	5 dpi	9 dpi	16 dpi	23 dpi
75A:258 (*Phr1*, *phtjR*)	Repression	83	24	36	86	153
	Induction	259	672	156	418	691
Wonga (*phr1*, *PhtjR*)	Repression	65	132	418	- ^2^	-
	Induction	364	534	1166		
Emir (*phr1*, *phtjR*)	Repression	53	26	208	-	-
	Induction	101	230	1496		

^1^ Days post inoculation. ^2^ Data point not analyzed.

**Table 3:** Biological process ontology terms that were significantly overrepresented in *Lupinus angustifolius* resistant (75A:258, Wonga) and susceptible (Emir) lines in their transcriptomic response to *Diaporthe toxica* inoculation.

Line.	75A:258			Wonga			Emir		
Days Post Inoculation	1 ^1^	5	9	1	5	9	1	5	9
Defense response	2.4 ^2^	>3	>3	2.7	>3	>3		>3	>3
Response to stress		>3	>3		>3	>3		>3	1.8
Oxidation-reduction process	>3	>3	2.7			>3			>3
Antibiotic catabolic process		>3	>3			>3			>3
Cofactor catabolic process		2.7	>3			3.0			>3
Drug catabolic process		2.7	>3			3.0			>3
Hydrogen peroxide catabolic process		>3	>3			>3			>3
Hydrogen peroxide metabolic process		>3	>3			>3			>3
Nucleic acid-templated transcription		2.2		1.9		2.5			2.0
Reactive oxygen species metabolic process		>3	>3			>3			>3
Regulation of biosynthetic process		2.7		2.5		>3			>3
Regulation of cellular biosynthetic process		2.7		2.5		>3			>3
Regulation of cellular macromolecule biosynthetic process		3.0		3.0		>3			>3
Regulation of cellular metabolic process		1.8		1.7		2.5			2.0
Regulation of gene expression		2.2		2.5		2.5			2.1
Regulation of macromolecule biosynthetic process		2.7		2.7		>3			>3
Regulation of macromolecule metabolic process		1.4		1.7		1.9			1.5
Regulation of nitrogen compound metabolic process		2.2		1.9		3.0			2.5
Regulation of nucleic acid-templated transcription		3.0		>3		>3			>3
Regulation of nucleobase-containing compound metabolic process		2.7		2.7		>3			>3
Regulation of primary metabolic process		2.0		1.8		3.0			2.0
Regulation of RNA biosynthetic process		3.0		>3		>3			>3
Regulation of RNA metabolic process		2.7		3.0		>3			>3
Regulation of transcription, DNA-templated		3.0		>3		>3			>3
Response to oxidative stress		1.7	3.0			3.0			1.7
RNA biosynthetic process		2.2		1.9		2.5			1.9
Transcription, DNA-templated		2.2		2.0		2.5			1.9
Antibiotic metabolic process			2.7			1.8			2.0
Oxylipin biosynthetic process	>3			>3					
Oxylipin metabolic process	>3			>3					
Regulation of metabolic process				1.4		1.5			
Response to drug						2.2			2.7
Response to stimulus					1.8	1.5			

^1^ Days post inoculation (dpi). ^2^ log10(FWER) for the over-representation of the Gene Ontology term.

**Table 4:** Molecular function ontology terms that were significantly overrepresented in *Lupinus angustifolius* resistant (75A:258, Wonga) and susceptible (Emir) lines in their transcriptomic response to *Diaporthe toxica* inoculation.

Line	75A:258			Wonga			Emir		
Days Post Inoculation	1 ^1^	5	9	1	5	9	1	5	9
Chitin binding		>3 ^2^	2.7		>3	1.5		>3	1.4
Heme binding	2.3	>3	>3		2.1	>3			>3
Oxidoreductase activity	>3	>3	>3		2.2	>3			>3
Sequence-specific DNA binding		1.4		>3	>3	>3		2.5	>3
Tetrapyrrole binding	2.2	>3	>3		1.6	>3			>3
DNA-binding transcription factor activity		>3		>3	>3	>3			>3
Transcription regulator activity		>3		>3	>3	>3			>3
Antioxidant activity		>3	2.7			>3			>3
Cofactor binding		1.4	1.6			>3			3.0
DNA binding		2.5		2.7		>3			>3
Endopeptidase inhibitor activity	3.0	2.7			3.0	2.3			
Endopeptidase regulator activity	3.0	2.7			3.0	2.3			
Oxidoreductase activity, acting on peroxide as acceptor		>3	3.0			>3			>3
Peptidase inhibitor activity	3.0	2.7			3.0	2.3			
Peptidase regulator activity	3.0	2.4			2.4	1.9			
Peroxidase activity		>3	3.0			>3			>3
Serine-type endopeptidase inhibitor activity		3.0			3.0	1.4			1.3
Catalytic activity						>3	1.7		>3
Dioxygenase activity	>3			1.7		2.7			
Electron transfer activity		1.6	3.0		3.0				

^1^ Days post inoculation (dpi). ^2^ log10(FWER) for the over-representation of the Gene Ontology term.

**Table 5:** RNAseq-based expression profiles of selected genes that were responsive to *Diaporthe toxica* inoculation.

Gene	75A:258					Wonga			Emir		
	1 ^1^	5	9	16	23	1	5	9	1	5	9
*TanjilG_24849*	5.8 ^2^	9.3	7.7	11.9	8.9	5.2	6.4	10.3	5.9	6.9	10.2
*TanjilG_24253*	5.2 ^3^	7.4	4.0	18.9	23.5	6.2	5.9	9.5	8.1	20.0	9.5
*TanjilG_05213*	2.9	6.5	3.7	4.1	5.0	4.1	4.7	5.0	1.5	3.0	7.8
*TanjilG_08982*	−2.0	20.7	18.4	- ^4^	17.3	21.0	21.1	7.2	17.6	-	23.1
*TanjilG_19904*	2.6	4.5	3.9	3.5	4.4	3.0	3.3	9.0	2.3	3.3	7.7
*TanjilG_10317*	2.0	4.5	3.2	3.2	3.8	2.5	2.4	6.3	3.2	2.9	6.3
*TanjilG_13015*	2.4	4.2	2.8	3.5	3.7	2.6	3.5	5.5	2.9	3.9	8.1
*TanjilG_27897*	4.7	24.8	24.0	19.4	23.5	4.9	25.0	27.5	22.1	21.3	11.1
*TanjilG_02482*	18.0	23.8	23.8	-	25.9	-	22.7	27.7	21.4	22.8	28.4
*TanjilG_23505*	3.5	4.7	3.9	6.4	6.8	3.7	3.2	8.1	1.7	4.7	6.8
*TanjilG_15237*	3.9	6.8	24.7	22.5	7.0	7.6	8.0	28.1	2.6	3.5	25.8
*TanjilG_10302*	15.4	42.7	3.1	38.3	40.8	36.8	5.4	8.0	2.7	38.4	9.4

^1^ Days post inoculation (dpi). ^2^ log_2_(fold change) of gene expression in inoculated plants as compared to control plants. ^3^ White background is used to highlight non-significant values. ^4^ Not expressed in both variants (inoculated and control plants).

**Table 6:** Real-time PCR-based expression profile of selected genes that are responsive to *Diaporthe toxica* inoculation.

Gene	75A:258					Wonga					Emir				
	1 ^1^	5	9	16	23	1	5	9	16	23	1	5	9	16	23
*TanjilG_24849*	10.1 ^2^	11.3	8.4	8.6	8.4	4.2	9.9	10.9	9.2	8.2	4.6	9.1	10.2	12.5	11.4
*TanjilG_24253*	8.1	9.3	5.2	5.7	5.6	−0.7	6.0	11.1	8.8	7.7	1.9	5.4	11.8	12.6	11.6
*TanjilG_05213*	3.7	8.4	5.2	5.8	4.9	4.4	5.2	8.2	3.5	3.7	2.4	4.3	8.7	8.7	8.2
*TanjilG_08982*	0.7	0.6 ^3^	0.2	−0.4	0.0	0.3	2.2	3.0	1.4	1.4	0.4	2.1	3.6	3.9	2.1
*TanjilG_19904*	2.3	2.8	2.2	3.4	3.0	5.3	4.6	7.6	5.8	6.4	1.1	2.6	5.7	7.5	6.5
*TanjilG_10317*	2.1	4.3	2.8	1.4	2.4	1.1	1.7	6.2	5.6	5.8	1.7	1.5	4.1	5.7	6.4
*TanjilG_13015*	3.5	5.1	3.5	1.7	3.1	3.1	4.8	7.6	3.6	4.6	1.1	3.4	6.7	6.7	6.9
*TanjilG_27897*	7.1	7.8	7.8	4.0	6.1	6.8	10.0	10.5	10.2	10.6	2.0	3.4	10.6	11.7	12.4
*TanjilG_02482*	1.7	1.4	0.1	9.4	3.3	−0.8	1.7	4.2	1.6	0.6	−1.0	0.6	7.2	3.1	1.9
*TanjilG_23505*	6.5	4.3	4.6	3.8	5.1	4.2	4.1	8.2	6.1	5.4	2.9	4.6	6.9	6.6	8.4
*TanjilG_15237*	8.2	9.0	8.9	3.6	5.3	7.8	9.7	14.4	11.8	8.3	4.8	4.3	9.5	15.5	11.9
*TanjilG_10302*	6.3	7.9	6.1	8.7	4.7	3.8	7.8	8.5	5.2	6.1	2.9	5.6	10.2	10.7	8.9

^1^ Days post inoculation (dpi). ^2^ log_2_(fold change) of gene expression in inoculated plants as compared to the control plants. ^3^ White background was used to highlight non-significant values.

## Data Availability

RNA-seq data were deposited in the ArrayExpress database [170] under accession number E-MTAB-9814.

## Supplementary Materials

The following are available online at https://www.mdpi.com/1422-0067/22/2/574/s1, Table S1: RAPD primers selected for the analysis of *Diaporthe toxica* isolates and the list of polymorphic bands obtained, Table S2: RNA concentration, RNA quality parameters and number of reads obtained from RNA sequencing, Table S3: Typical domains identified using Disease Resistance Analysis and Gene Orthology (DRAGO 2) tool at Plant Resistance Genes database (PRGdb), Table S4: Overrepresented gene ontology terms identified for the sets of differentially expressed genes, Table S5: Results of weighted gene co-expression network analysis, Table S6: Overrepresented gene ontology terms in modules identified by weighted gene co-expression network analysis, Table S7: Overrepresented gene ontology terms identified for the sets of genes which were significantly upregulated only in 75A:258 or in Wonga, Table S8: Results of differential gene expression profiling, Table S9: Results of gene expression profiling by quantitative PCR, Table S10: RNA concentration and A260/A280 ratio of samples prepared for gene expression profiling by quantitative PCR, Table S11: Designed primers and expected PCR product sizes for gene expression profiling by quantitative PCR, Table S12: Calculated values of PCR efficiency and R-squared for gene expression profiling by quantitative PCR.

## Abbreviations

cv.	cultivar
DIR1	defective in induced resistance 1
dpi	day post inoculation
GST	glutathione S-transferase
ITS	internal transcribed spacer
*Phr1*	allele conferring *D. toxica* resistance in the 75A:258 *L. angustifolius* line
*Phr2*	allele conferring *D. toxica* resistance in the *L. angustifolius* cultivar Merrit
*PhtjR*	allele conferring *D. toxica* resistance in the *L. angustifolius* cultivar Wonga
RAPD	random amplification of polymorphic DNA
SAM22	starvation-associated message 22
SAR	systemic acquired resistance
WGCNA	weighted gene co-expression network analysis
XTH	xyloglucan endotransglucosylase/hydrolase
